# Ultrasound measurements of fetal facial profile markers and their associations with congenital malformations during early pregnancy

**DOI:** 10.1186/s12884-023-06067-6

**Published:** 2023-11-04

**Authors:** Minyan Liao, Limin Wang, Ning Shang, Xueyi Hu, Bingjia He, Xiangjiao Liu, Guanghua Xiang, Wei Zhong

**Affiliations:** 1 Guangdong Women and Children Hospital, 521 Xing-nan Avenue Pan-Yu, Guangzhou, 510499 China; 2 Second Affiliated Hospital of Guangzhou Medical University, Guangzhou, 510260 China

**Keywords:** Pregnancy, Facial profile, Congenital malformations, Sensitivity, Specificity

## Abstract

**Background:**

Fetal facial profile could be measured during the early pregnancy. Its abnormalities might be associated with certain congenital malformations. We aimed to study the associations between fetal facial profile measurements with crown-rump length and congenital malformations (cleft lip and palate, micrognathia, and open spina bifida) during early pregnancy.

**Methods:**

We performed a prospective cross-sectional study between June 2019 and April 2022. Pregnant women at a gestational age between 11–13^+ 6^ weeks were enrolled. Two sonographers performed fetal facial profile measurements independently. The associations between these measurements with crown-rump length and congenital malformations were evaluated.

**Results:**

There were 406 and 25 fetuses without or with congenital malformations, respectively. Two sonographers showed satisfactory inter- and intra-observer agreements and reproducibility. The maxillary gap was only observed in 7.6% of normal fetuses, whereas all cleft lip and palate fetuses had a maxillary gap ≥ 0.8 mm. The crown-rump length was negatively correlated with frontomaxillary facial angle, inferior facial angle, and profile line distance but positively correlated with maxilla-nasion-mandible angle, facial maxillary angle, frontal space distance, and palatine maxillary diameter. These measurements showed various significant changes with different congenital malformations.

**Conclusions:**

Measurements of fetal facial profile in early pregnancy were feasible with satisfactory reproducibility. These measurements correlated with crown-rump length and showed significant differences with certain fetal congenital malformations.

**Supplementary Information:**

The online version contains supplementary material available at 10.1186/s12884-023-06067-6.

## Background

Fetal craniofacial malformations refer to a diverse group of growth deformities in the head and facial areas in the fetus [1]. It can cause various degrees of disfigurements, leading to developmental and functional abnormalities [2, 3]. Studies also found that craniofacial malformations can associate with more serious chromosomal abnormalities or metabolic disorders [4, 5]. Early identifications of craniofacial malformations can facilitate informed prenatal counseling, appropriate genetic evaluations, and careful family planning.

Ultrasound is the main diagnostic test used to assess fetal structural malformations [6]. Most studies have described the utilities of prenatal facial anatomy determination by ultrasound scanning starting from the middle trimester [7]. With the advances in ultrasound technology, some craniofacial structures could be evaluated during the first trimester of pregnancy. The International Society of Ultrasound in Obstetrics and Gynecology (ISUOG) has issued a guideline recommending ultrasound scans between 11 and 13^+ 6^ weeks [8]. Certain measurements, such as nasal bone length, the ratio of anterior nasal tissue thickness to nasal bone, and fourth ventricle size, were applied in the early prenatal screening to look for structure and potential chromosome abnormalities [9–11]. However, there were limited studies investigating the relationship between craniofacial malformation and other congenital malformations during early pregnancy. These studies were especially sparse in China, despite several research groups already reporting racial differences in the normal facial profile marker measurements [12, 13]. Understanding the facial profile marker measurements and their associations with other congenital abnormalities could help decide treatments.

Therefore, we performed the present prospective cross-sectional study. First, we determined the agreement and reproducibility of fetal facial profile measurements by ultrasound during early pregnancy. Then, we evaluated the associations between these measurements and crown-rump length (CRL) and congenital malformations. We aimed to further provide a preliminary normal measurement reference for the Chinese population and explore the clinical implications of these abnormal measurements.

## Methods

### Study design and participant selection

We performed a prospective cross-sectional study on pregnant women at the Guangdong Women and Children Hospital, China, between June 2019 and April 2022. The study protocol was approved by the hospital ethics committee. All the study participants signed the written informed consent. The inclusion criterion was Chinese women who (1) had singleton pregnancy at the gestational age between 11 weeks and 13^+ 6^ weeks; (2) were willing to receive prenatal ultrasound evaluations with clear images obtained. We targeted to study the fetuses with normal facial profile measurements or cleft lip and palate, micrognathia, or open spina bifida. Fetuses with other structural abnormalities were excluded from the study. In addition, fetuses with abnormal chromosome analysis were also excluded since these fetuses might have unique facial profiles, which was not the purpose of the present study.

### Ultrasound equipment

Ultrasound equipment included GE Voluson E10, E8, and E6 and Samsung HERA W9 and Aplio i700. They were equipped with a 2-dimensional (2D) convex probe with a frequency of 2.0–5.0 mHz, 6.0–8.0 mHz, and 3.0–10.0 mHz.

### Ultrasound examinations

The ultrasound examinations followed the guidelines from ISUOG [8]. Briefly, the eligible pregnant women were placed supine on the stretcher. All the fetal facial profiles were measured through the transabdominal ultrasound examinations. When evaluating congenital malformations, we used the transabdominal ultrasound approach first, with transvaginal approach if necessary. The measurements included the frontomaxillary facial (FMF) angle, maxilla-nasion-mandible (MNM) angle, facial maxillary angle (FMA), inferior facial angle (IFA), frontal space distance (FSD), profile line (PL) distance, palatine maxillary diameter (PMD), and maxillary gap (MG). These measurements were performed by two licensed sonographers. Each sonographer performed two rounds of measurements one week apart independently. In each round, the sonographers performed the measurements twice. The average of these two measurements was taken for the analysis. Therefore, two measurements were carried out for each facial profile marker from each sonographer in these two rounds of measurements. In order to make sure that we obtained images with adequate qualities, we spent a minimum 8 min for ultrasound measurements in each individual patient.

**Fig. 1:**
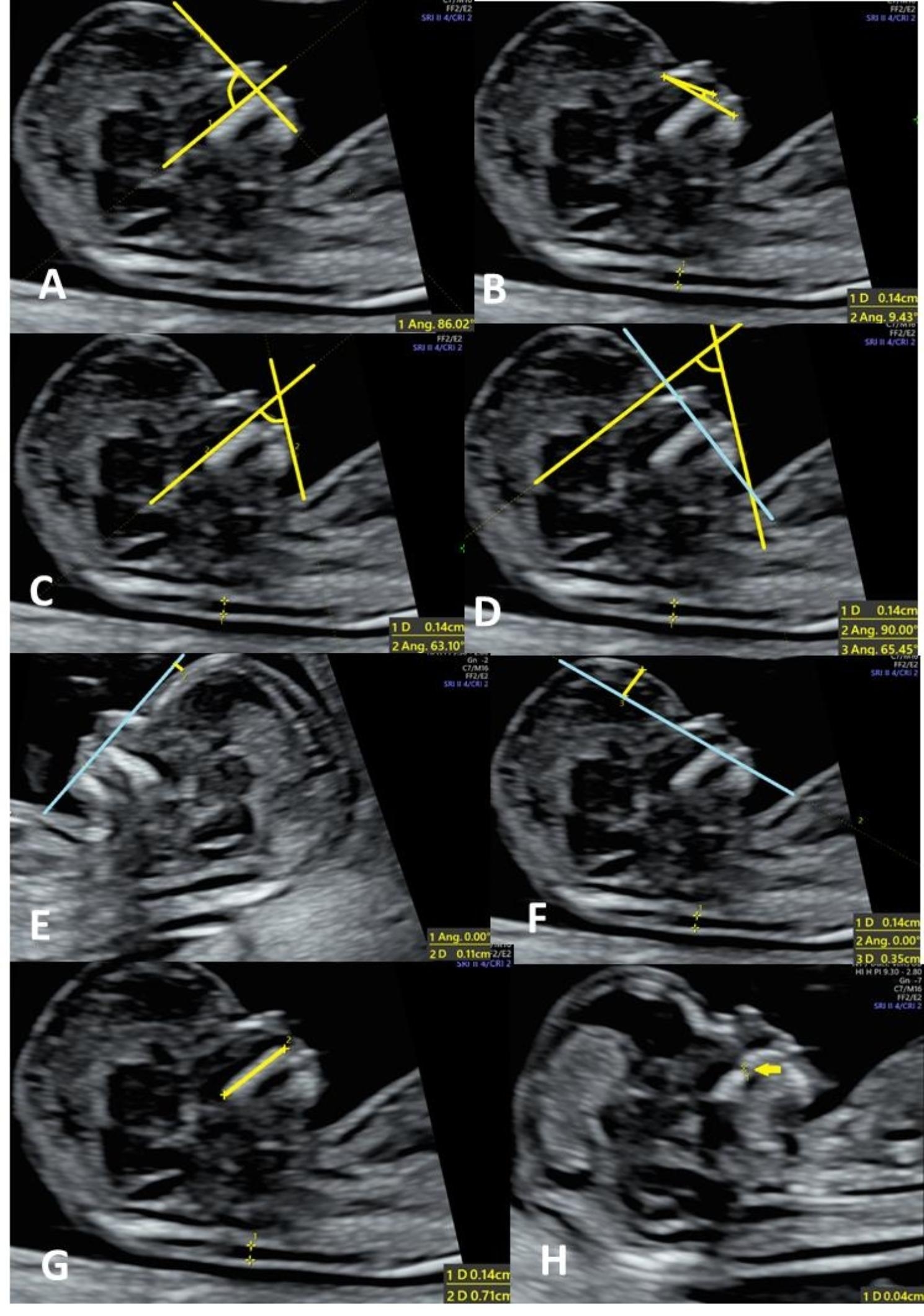
Measurements of different facial profile markers. **A**, frontomaxillary facial angle (FMF, 12^+ 5^ weeks); **B**, maxilla-nasion-mandible angle (MNM, 12^+ 5^ weeks); **C**, facial maxillary angle (FMA, 12^+ 5^ weeks); **D**, inferior facial angle (IFA, 12^+ 5^ weeks); **E**, frontal space distance (FSD, 13^+ 5^ weeks); **F**, profile line distance (PL distance, 12^+ 5^ weeks); **G**, palatine maxillary diameter (PMD, 12^+ 5^ weeks); **H**, maxillary gap (MG, 12^+ 5^ weeks). **A**, FMF angle: the angle between the extension line along the superior edge of the palate and the tangent line of the frontal bone on the mid-sagittal plane with the front end of the palate as the apex [14]. **B**, MNM angle: the angle between the maxilla–nasion line and the mandible–nasion line. The nasion was defined as the most anterior point at the intersection of the frontal and nasal bone [15]. **C**, FMA: the angle between the line overlying the maxilla and the line across the mentum tip and upper lip [16]. **D**, IFA: the angle between the line orthogonal to the vertical part of the forehead at the level of the synostosis of the nasal bones and a second line joining the tip of the mentum to the anterior point of the more protruding lip [17]. **E**, FSD: the maximum perpendicular distance from the mandibulo-maxillary line (MML) to the most prominent part of the fetal forehead. The MML was an extended line intersecting the most anterior portions of the mandible and the maxilla [18]. **F**, PL distance: the maximum perpendicular distance from the facial profile line (FPL) to the outer border of the forehead. The FPL was the line passing through the middle point of the anterior border of the mandible and the nasion [19]. **G**, PMD: the shortest hyperechoic distance from the ossified posterior palatine process to the anterior ossified part of the maxilla [20]. **H**, MG: if present, the size of the gap was measured [21]

The definitions for the facial profile markers are described in Fig. 1.

In addition, we also measured the CRL, which was the length from the top of the head to the bottom of the buttocks.

### Statistical analysis

The intra- and inter-observer agreements and reproducibility were assessed by the intraclass correlation coefficient (ICC) and Bland-Altman analysis.

Based on the absence or presence of congenital malformations (cleft lip and palate, micrognathia, or open spina bifida), [8] these fetuses were assigned into either the normal group or abnormal group. The differences in the facial profile markers between these two group fetuses were compared by the Mann-Whitney U test. The relationships between each facial profile marker with the CRL, cleft lip and palate, micrognathia, and open spina bifida were examined by the Pearson correlation analysis. All the statistical analyses were performed in SPSS (version 19.0, IBM, New York, USA) and Med calc software. A *P* < 0.05 was considered statistically significant.

## Results

### Participant enrollments and facial profile markers

A total of 542 pregnant women received fetal ultrasound evaluations during early pregnancy. Among them, 2, 4, 3, and 1 fetuses had thickened nuchal translucency, torso abnormalities, demise, and maternal uterus malformation, respectively, and were excluded. In addition, 76 and 21 fetuses had no clear mid-sagittal plane or facial profile images, respectively, and were excluded. Four fetuses were excluded due to chromosome abnormalities (2 cases of trisomy 13 and 2 cases of trisomy 18). Finally, there were 431 fetuses included in the analysis, with 406 fetuses in the normal group and 25 in the abnormal group (seven bilateral cleft lip and palate, seven unilateral cleft lip and palate, seven micrognathia, and four open spina bifida). Transabdominal ultrasound examinations were able to obtain satisfactory images in all pregnant women in the normal group. Four pregnant women in the abnormal group (16%) had to receive additional transvaginal ultrasound examination to obtain clear images. All the pregnant women with abnormal fetuses selected induced abortion (at an average gestational week of 15^+ 2^ weeks). The final congenital abnormal structure diagnoses were confirmed on the fetal autopsy.

The age of the pregnant women and the CRL of the fetuses had no statistically significant differences between the normal and abnormal groups (30.0 ± 4.3 versus 29.0 ± 5.6 years old, *P* = 0.372, 61.0 ± 6.7 versus 66.0 ± 6.8 mm, *P* = 0.384, respectively). In the normal group, the FMF angle, MNM angle, FMA, IFA, FSD, PL distance, and PMD were 86.4°±3.1°, 7.8°±2.1°, 64.3°±3.8°, 67.1°±3.5°, -1.7 ± 1.6 mm, 3.4 ± 0.6 mm, and 6.6 ± 0.9 mm, respectively. MG was only observed in 7.6% of fetuses without congenital malformations, whereas all fetuses with cleft lip and palate had an MG ≥ 0.8 mm. Detailed measurements of fetal facial profile markers in the normal and abnormal groups are provided in the supplementary materials (Supplementary Table S1 and S2).

### Intra- and inter-observer agreements and reproducibility

All the facial profile markers showed satisfactory intra- and inter-observer agreements and reproducibility (Tables 1 and 2).

**Table 1:** Intra- and inter-observer agreements on the facial profile marker measurements

	Observer 1				Observer 2			Observer 1 and 2	
	Mean	95% LoA			Mean	95% LoA		Mean	95% LoA
FMF	0.169	(-2.731 ~ 3.069)			0.059	(-1.094 ~ 1.212)		0.034	(-1.835 ~ 1.903)
MNM	-0.156	(-1.743 ~ 1.432)			-0.003	(-0.983 ~ 0.976)		-0.201	(-1.425 ~ 1.022)
FMA	-0.179	(-2.742 ~ 2.383)			-0.038	(-0.763 ~ 0.686)		-0.194	(-2.857 ~ 2.469)
IFA	-0.083	(-2.655 ~ 2.490)			-0.011	(-0.762 ~ 0.740)		-0.189	(-4.273 ~ 3.896)
FSD	-0.008	(-0.236 ~ 0.219)			0.023	(-0.227 ~ 0.274)		-0.079	(-0.623 ~ 0.464)
PL	-0.043	(-0.283 ~ 0.196)			-0.063	(-0.658 ~ 0.531)		0.010	(-0.409 ~ 0.429)
PMD	-0.023	(-0.240 ~ 0.193)			0.0467	(-0.209 ~ 0.303)		-0.094	(-0.591 ~ 0.403)

FMA, facial maxillary angle; FMF, frontomaxillary facial angle; FSD, frontal space distance; IFA, inferior facial angle; LoA, limits of agreement; MNM, maxilla-nasion-mandible angle; PL, profile line distance; PMD, palatine maxillary diameter

**Table 2:** Intra- and inter-observer reproducibility on the facial profile marker measurements

	Observer 1 ICC (95% CI)	Observer 2 ICC (95% CI)	Observer 1 and 2 ICC (95% CI)
FMF	0.897(0.896 ~ 0.915)	0.952(0.902 ~ 0.977)	0.885(0.773–0.944)
MNM	0.906(0.886 ~ 0.922)	0.943(0.884 ~ 0.997)	0.888(0.778–0.945)
FMA	0.933(0.920 ~ 0.945)	0.870(0.746 ~ 0.936)	0.830(0.673–0.915)
IFA	0.909(0.890 ~ 0.924)	0.938(0.874 ~ 0.970)	0.812(0.642–0.906)
FSD	0.942(0.930 ~ 0.952)	0.994(0.987 ~ 0.997)	0.961(0.919–0.981)
FPL	0.979(0.975 ~ 0.983)	0.878(0.759 ~ 0.940)	0.887(0.776–0.944)
PMD	0.979(0.974 ~ 0.983)	0.879(0.956 ~ 0.990)	0.879(0.762–0.941)

CI, confidence interval; FMA, facial maxillary angle; FMF, frontomaxillary facial angle; FSD, frontal space distance; ICC, intraclass correlation coefficient; IFA, inferior facial angle; MNM, maxilla-nasion-mandible angle; PL, profile line distance; PMD, palatine maxillary diameter

### Correlations between CRL and facial profile markers

**Fig. 2:**
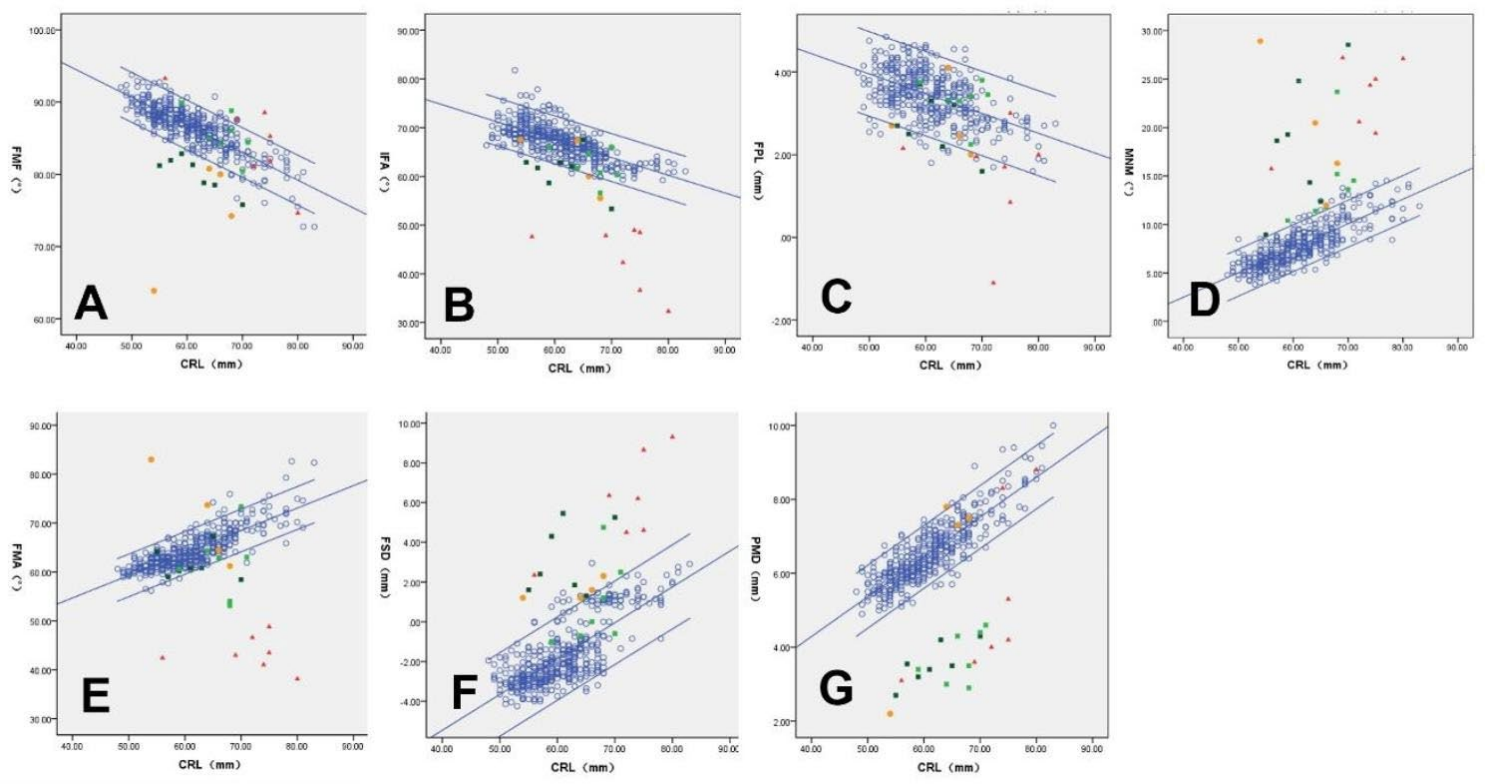
Correlations between CRL and facial profile markers. CRL was negatively correlated with the FMF angle (A, *R²*= 0.685, *P* < 0.001), IFA angle (B, *R*^*2*^ = 0.482, P < 0.001), and PL distance (C, *R*^*2*^ = 0.278, *P* < 0.001), but positively correlated with the MNM angle (D, *R*^*2*^ = 0.660, *P* < 0.001), FMA angle (E, *R*^*2*^ = 0.656, *P* < 0.001), FSD (F, *R*^*2*^ = 0.563, *P* < 0.001), and PMD (G, *R*^*2*^ = 0.737, *P* < 0.001). FMA, facial maxillary angle; FMF, frontomaxillary facial angle; FSD, frontal space distance; IFA, inferior facial angle; MNM, maxilla-nasion-mandible angle; PL, profile line distance; PMD, palatine maxillary diameter. Bilateral cleft lip and palate (dark green square), unilateral cleft lip and palate (light green square), micrognathia (red triangle), open spina bifida (orange circle), and normal controls (blue hollow circle)

CRL was negatively correlated with the FMF angle, IFA angle, and PL distance but positively correlated with the MNM angle, FMA angle, FSD, and PMD (Fig. 2).

### Changes of facial profile markers with congenital malformations

As shown in Table 3, the FMF angle significantly decreased in fetuses with bilateral cleft lip and palate or open spina bifida. The MNM angle significantly increased in fetuses with cleft lip and palate, micrognathia, or open spina bifida. The FMA significantly decreased in fetuses with cleft lip and palate or micrognathia. The IFA significantly decreased in fetuses with cleft lip and palate, micrognathia, or open spina bifida. The FSD significantly increased in fetuses with bilateral cleft lip and palate, micrognathia, or open spina bifida. The PL distance significantly decreased in fetuses with bilateral cleft lip and palate micrognathia. The PMD significantly decreased in fetuses with cleft lip and palate.

**Table 3:** Changes of fetal facial profile markers in fetuses with congenital malformations

Measures	Normal group (N = 406)	Abnormal group (N = 25)		*P*	Sensitivity	Specificity
FMF	0.021(3.100)	Bilateral cleft lip and palate	-6.189(2.461)	< 0.0001	100% (7/7)	98.8% (401/406)
		Unilateral cleft lip and palate	1.295(3.137)	> 0.05		
		Micrognathia	2.212(6.038)	> 0.05		
		Open spina bifida	-10.929(7.794)	< 0.001	100% (4/4)	98.8% (401/406)
MNM	-0.006(2.096)	Bilateral cleft lip and palate	10.208(6.909)	< 0.0001	100% (7/7)	96.8% (393/406)
		Unilateral cleft lip and palate	5.158(4.451)	< 0.0001	100% (7/7)	96.8% (393/406)
		Micrognathia	12.285(4.299)	< 0.0001	100% (7/7)	96.8% (393/406)
		Open spina bifida	11.099(7.209)	< 0.001	100% (4/4)	96.8% (393/406)
FMA	0.004(3.791)	Bilateral cleft lip and palate	-3.011(3.140)	< 0.001	14.3% (1/7)	98.5% (400/406)
		Unilateral cleft lip and palate	-5.266(6.830)	< 0.05	42.9% (3/7)	98.5% (400/406)
		Micrognathia	-25.833(3.511)	< 0.0001	100% (7/7)	98.5% (400/406)
		Open spina bifida	5.346(9.797)	> 0.05		
IFA	-0.020(3.494)	Bilateral cleft lip and palate	-5.727(4.375)	< 0.001	57.1% (4/7)	99.5% (404/406)
		Unilateral cleft lip and palate	-2.858(3.435)	< 0.05	14.3% (1/7)	99.5% (404/406)
		Micrognathia	-19.868(6.643)	< 0.0001	100% (7/7)	99.5% (404/406)
		Open spina bifida	-3.836(5.891)	< 0.05	50% (2/4)	99.5% (404/406)
FSD	0.023(1.613)	Bilateral cleft lip and palate	4.785(1.784)	< 0.0001	100% (7/7)	97.8% (397/406)
		Unilateral cleft lip and palate	1.574(2.109)	< 0.05	28.6% (2/7)	97.8% (397/406)
		Micrognathia	5.788(2.436)	< 0.0001	100% (7/7)	97.8% (397/406)
		Open spina bifida	2.913(0.519)	< 0.001	100% (4/4)	97.8% (397/406)
PL distance	0.013(0.608)	Bilateral cleft lip and palate	-0.650(0.718)	< 0.05	42.9% (3/7)	96.8% (393/406)
		Unilateral cleft lip and palate	0.176(0.514)	> 0.05		
		Micrognathia	-1.399(1.313)	< 0.001	71.4% (5/7)	96.8% (393/406)
		Open spina bifida	-0.505(0.906)	> 0.05		
PMD	-0.016(0.843)	Cleft lip and palate	-3.316(0.741)	< 0.0001	100% (20/20)	97.8% (397/406)
MG	-0.009(0.073)	Cleft lip and palate	1.001(0.682)	< 0.0001	100% (20/20)	92.4% (375/406)

FMA, facial maxillary angle; FMF, frontomaxillary facial angle; FSD, frontal space distance; IFA, inferior facial angle; MG, maxillary gap; MNM, maxilla-nasion-mandible angle; PL, profile line distance; PMD, palatine maxillary diameter

The abnormal facial profile marker measurements were shown in the supplementary Figure S1. Figure S2 shows the congenital malformations in fetuses during autopsy after the induced labor.

## Discussion

In the present study, we provided fetal facial profile measurements in the Chinese population during early pregnancy. We showed satisfactory agreements between different sonographers and sufficient reproducibility between different measurements. In addition, we further studied the relationships between the fetal facial profile measurements with CRL and certain congenital malformations, which had some new findings.

### FMF angle

Our study showed that 74% of normal fetuses had an FMF angle larger than 85.0°. We also found a negative correlation between the FMF angle and CRL, which was consistent with a study from Borenstein et al [14]. The FMF angle was reduced in fetuses with open spina bifida, with a statistically significant difference from the normal fetuses (100% sensitivity). Overall, the FMF angle could help the diagnosis of fetal bilateral cleft lip and palate and open spina bifida with 100% sensitivity and 98.8% specificity.

### MNM angle

In the present study, the mean MNM angle was 7.8 (± 2.1)°, which had some variations from the reports by the other two studies in Chinese populations (Ji et al. and Liu et al. reported 1.84–6.50° and 11.00°±2.58, respectively) [19, 22]. We also found a positive correlation between MNM angle and CRL, which was consistent with the studies by Ji et al. and Bakker et al., [9, 19] but different from the result from Liu et al [22]. Our study found an increased MNM angle in fetuses with cleft lip and palate, micrognathia, and open spina bifida, similar to a previous study [9]. Therefore, the MNM angle can help the diagnosis of cleft lip and palate, micrognathia, and open spina bifida with a sensitivity of 100% and specificity of 96.8%.

### FMA

We measured the FMA with the anterior surface of the maxilla as the reference line to avoid the influence of the vomer curvature [16]. The mean measurement of FMA was 64.3 (± 3.8)°, positively correlated with CRL, which was consistent with the reports from Ji et al [19]. In our study, we used an FMA of 50° as the cut-off value for the diagnosis of micrognathia because all fetuses with a micrognathia had an FMA < 50°. The sensitivities of a decreased FMA for detecting micrognathia, bilateral cleft lip and palate, and unilateral cleft lip and palate fetuses were 100%, 14.3%, and 42.7%, respectively, with a specificity of 98.5%. Therefore, FMA could help the diagnosis of micrognathia and cleft lip and palate, especially for the former with high sensitivity and specificity.

### IFA

Our present study found an IFA of 67.1 ± 3.5° in normal fetuses, which was smaller than that reported by Ji et al. (80.2 ± 7.3°),^15^ but similar to that reported by Li et al. (66.5°) [23]. We also found a negative correlation between IFA and CRL, which was similar to the report by Tekesin et al., Ji et al., and Orzechowski et al., [19, 24, 25] but different from the report from Li et al [23]. In the present study, the sensitivity of IFA reduction for detecting micrognathia, bilateral cleft lip and palate, unilateral cleft lip and palate, and open spina bifida was 100%, 57.1%, 14.3%, and 50%, respectively, with a specificity of 99.5%. Therefore, IFA can help the diagnosis of these malformations, especially for micrognathia diagnosis with high sensitivity and specificity.

### FSD

There was no previously reported FSD measurement in Chinese fetuses. Our FSD measurement was − 1.7 ± 1.6 mm, which was smaller than those from other countries (1.3 ± 1.2 mm in Netherlands and Germany, 0.76 ± 0.40 mm in Germany, and − 0.97 − 0.74 mm in the Netherlands) [9, 18, 24]. The sensitivities of an increased FSD in the diagnosis of micrognathia, bilateral cleft lip and palate, unilateral cleft lip and palate, and open spina bifida were 100%, 100%, 28.6%, and 100%, respectively, with the specificity of 97.8%. Therefore, FSD can help the diagnosis of micrognathia, cleft lip and palate, and open spina bifida, with high sensitivity and specificity for micrognathia, bilateral cleft lip and palate, and open spina bifida.

### PL distance

The mean PL distance in this study was 3.4 ± 0.6 mm, which was slightly higher than 2.8 ± 0.5 mm reported by Ji et al [19]. The PL distance was negatively correlated with CRL. The sensitivities of PL distance to diagnose micrognathia and bilateral cleft lip and palate were 71.4% and 42.9%, respectively, with a specificity of 96.8%. PL distance can be used to help the diagnosis of bilateral cleft lip and palate and micrognathia.

### PMD

The PMD measurement was never reported in Chinese fetuses previously. The PMD measured in the present study was 6.6 ± 0.9 mm. PMD was positively correlated with CRL. In 25 fetuses in the abnormal group in the present study, there were five fetuses with micrognathia and concurrent cleft lip and palate and one fetus with a case of open spina bifida and concurrent cleft lip and palate. A total of 20 fetuses with cleft lip and palate had reduced PMD, which was statistically significantly different from the normal group. None of the fetuses with normal PMD were found to have cleft lip and palate. The sensitivity and specificity of PMD for detecting cleft lip and palate were 100% and 97.8%, respectively. PMD can be used as a reliable measurement to assess the cleft lip and palate, with high sensitivity and specificity.

### MG

In 2015, Chaoui et al. proposed that MG could screen for cleft lip and palate [21]. They reported that MG was present in 96% of cleft lip and palate with additional defects, 65% of isolated cleft lip and palate, and 7% of normal fetuses. Our study observed MG in 7.6% of normal fetuses, all within 0.6 mm. MG above 0.8 mm was observed in all 20 fetuses with cleft lip and palate. The sensitivity and specificity of MG in the diagnosis of cleft lip and palate were 100% and 92.4%, respectively. An additional test to rule out the cleft lip and palate should be performed in fetuses with an MG ≥ 0.8 mm.

The limitations of the present study included its single-center research with a small number of abnormal cases. We used the 2D ultrasound in our fetal evaluations. It was suggested that three-dimensional ultrasound could provide a better mid-sagittal view for accurate measurements. Our measurements in the normal fetuses still had some variations from other reports conducted in the Chinese population, which might be due to body habitus differences in the different geographic areas of China. Future multicenter research is required.

## Conclusions

Measurements of fetal facial profile in early pregnancy were feasible with satisfactory reproducibility in Chinese pregnant women. These measurements correlated with crown-rump length and showed significant differences with certain fetal congenital malformations.

### Electronic supplementary material

Below is the link to the electronic supplementary material.

Supplementary Material 1

## Data Availability

The datasets used and/or analysed during the current study available from the corresponding author on reasonable request.

## Abbreviations

CRL: Crown-rump length
FMA: Facial maxillary angle
FMF: Frontomaxillary facial
FSD: Frontal space distance
ICC: Intraclass correlation coefficient
IFA: Inferior facial angle
ISUOG: The International Society of Ultrasound in Obstetrics and Gynecology
MG: Maxillary gap
MNM: Maxilla-nasion-mandible
PL: Profile line
PMD: Palatine maxillary diameter
