# Supplementary material for: Ultrasound measurements of fetal facial profile markers and their associations with congenital malformations during early pregnancy
Source: BMC Pregnancy Childbirth. 2023 Nov 4;23:772. doi: 10.1186/s12884-023-06067-6 (PMC10625258; doi:10.1186/s12884-023-06067-6)
Supplement: Supplementary file 1 — Supplementary Material 1 [file 12884_2023_6067_MOESM1_ESM.docx]

**Supplementary materials**

**Figures**


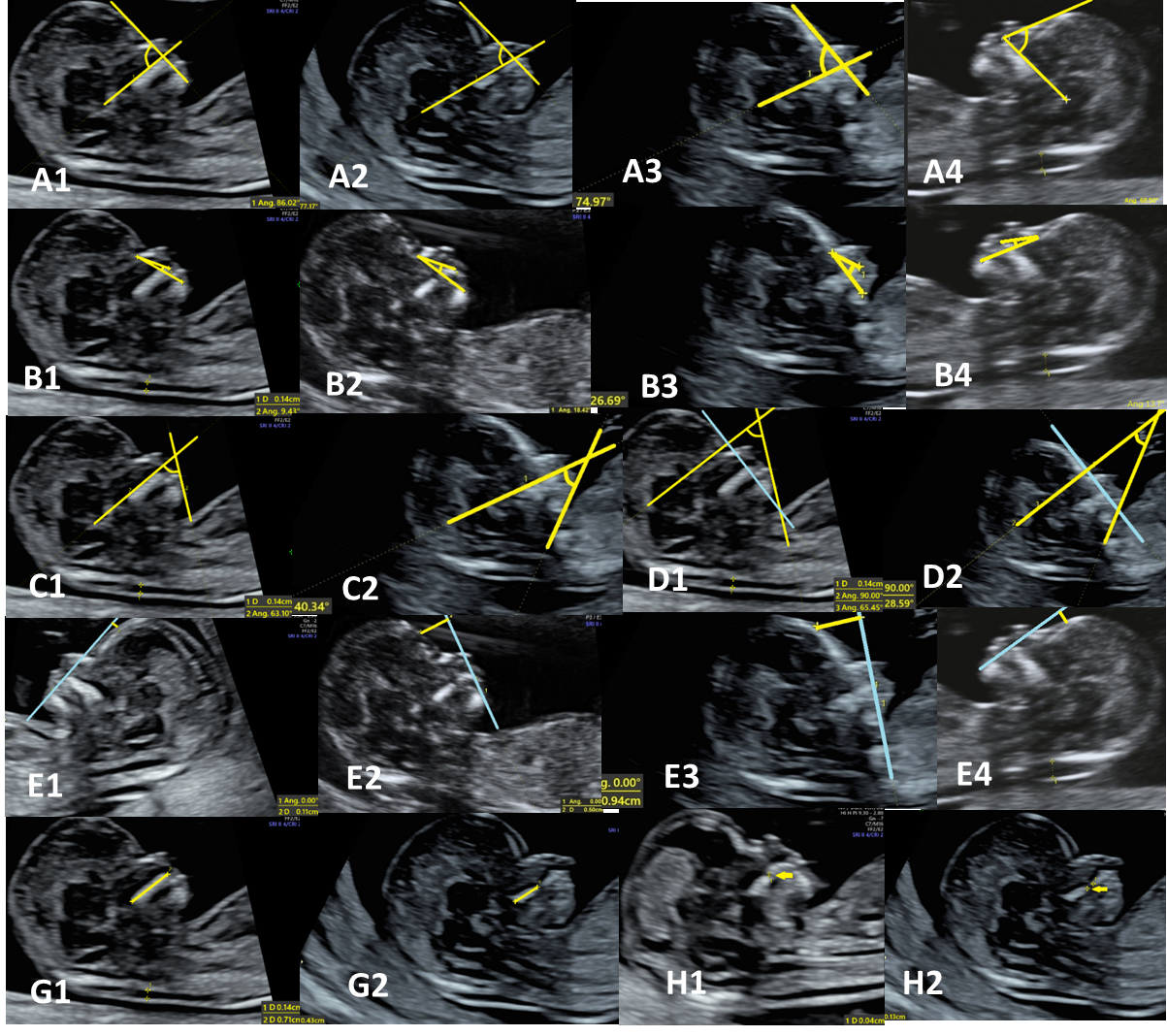


**Figure S1.** Normal and abnormal fetal facial profile marker measurements under prenatal ultrasound examinations. A1, B1, C1, D1, E1, G1, and H1 show normal fetal facial profiles. A2-4, B2-4, C2, D2, E2-4, G2, and H2 show abnormal fetal profiles as listed in the following Table.

| Measurements | | Gestational age, weeks | Measures |
| --- | --- | --- | --- |
| A, FMF | A1, normal | 12^+5^ | 86.02° |
|  | A2, bilateral cleft lip and palate | 13^+1^ | 77.17° |
|  | A3, micrognathia | 13^+6^ | 74.97° |
|  | A4, spina bifida | 13 | 68.50° |
| B, MNM | B1, normal | 12^+5^ | 9.43° |
|  | B2, unilateral cleft lip and palate | 12^+6^ | 18.42° |
|  | B3, micrognathia | 13^+6^ | 26.69° |
|  | B4, spina bifida | 13 | 13.70° |
| C, FMA | C1, normal | 12^+5^ | 63.10° |
|  | C2, micrognathia | 13^+6^ | 40.34° |
| D, IFA | D1, normal | 12^+5^ | 65.45° |
|  | D2, micrognathia | 13^+6^ | 28.59° |
| E, FSD | E1, normal | 13^+5^ | 1.1mm |
|  | E2, unilateral cleft lip and palate | 12^+5^ | 5.0mm |
|  | E3, micrognathia | 13^+6^ | 9.4mm |
|  | E4, spina bifida | 13 | 2.3mm |
| G, PMD | G1, normal  G2, bilateral cleft lip and palate | 12^+5^  13^+1^ | 7.1mm  4.3mm |
| H, MG | H1, normal | 12^+5^ | 0.4mm |
|  | H2, bilateral cleft lip and palate | 13^+1^ | 1.3mm |

FMA, facial maxillary angle; FMF, frontomaxillary facial angle; FSD, frontal space distance; IFA, inferior facial angle; MG, maxillary gag; MNM, maxilla-nasion-mandible angle; PL, profile line distance; PMD, palatine maxillary diameter.


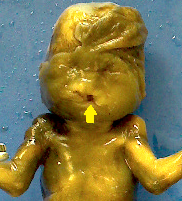

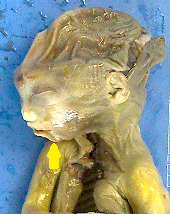


**B**

**A**

**Figure S2.** Fetal autopsy after induced abortion. A, Left cleft lip and palate in a fetus at 16 gestational weeks (corresponding to B2, E2 in Figure S1). B, Micrognathia in a fetus at 19^+3^ gestational weeks (corresponding to Figure A3, B3, C2, D2, and E3).

**Tables**

**Table S1.** Fetal facial profile measurements in the normal and abnormal groups.

| Groups | | FMF (°) | MNM (°) | FMA (°) | IFA (°) | FSD (mm) | PL(mm) | PMD (mm) |
| --- | --- | --- | --- | --- | --- | --- | --- | --- |
| Normal, CRL categories (N) | |  |  |  |  |  |  |  |
|  | 45-54(73) | 89.34(1.77) | 5.77(0.96) | 61.00(1.53) | 69.68(2.71) | -2.92(0.66) | 3.73(0.50) | 5.70(0.37) |
|  | 55-64(221) | 87.11(1.79) | 7.36(1.33) | 63.23(2.18) | 67.99(2.95) | -2.11(1.05) | 3.53((0.55) | 6.36(0.48) |
|  | 65-74(95) | 83.76(2.63) | 9.68(1.77) | 67.78(2.81) | 64.10(2.40) | -0.26(1.63) | 3.08(0.55) | 7.28(0.60) |
|  | 75-84(17) | 79.90(3.49) | 12.09(1.62) | 73.28(4.65) | 62.11(1.75) | 1.52(0.90) | 2.63(0.55) | 8.65(0.66) |
|  | Total (406) | 86.43(3.10) | 7.81(2.10) | 64.30(3.82) | 67.14(3.49) | -1.67(1.61) | 3.43(0.61) | 6.55(0.85) |
| Abnormal, types (N) | |  |  |  |  |  |  |  |
|  | Bilateral cleft lip and palate (7) | 80.06(2.46) | 18.13(6.91) | 61.50(3.14) | 61.28(4.38) | 3.16(1.78) | 2.74(0.72) | 3.55(0.56) |
|  | Unilateral cleft lip and palate (7) | 85.58(3.14) | 14.39(4.45) | 61.60(6.83) | 62.28(3.44) | 0.88(2.11) | 3.32(0.51) | 3.73(0.70) |
|  | Micrognathia (7) | 84.59(6.04) | 22.78 (4.30) | 43.34(3.51) | 43.45(6.64) | 5.99(2.44) | 1.51(1.31) | 5.33(2.30) |
|  | Open spina bifida (4) | 74.72(7.79) | 19.42(7.21) | 70.58(9.80) | 62.60(5.89) | 1.58(0.52) | 2.81(0.91) | 6.20(2.67) |

All measurements are presented as mean (standard deviation).

CRL, crown-rump length; FMA, facial maxillary angle; FMF, frontomaxillary facial angle; FSD, frontal space distance; IFA, inferior facial angle; MNM, maxilla-nasion-mandible angle; PL, profile line distance; PMD, palatine maxillary diameter.

**Table S2.** Maxillary gap measurements in the normal fetuses and fetuses with cleft lip and palate.

|  | CRL (mm) | N | MG, N(%) | |  | MG(mm) | | |
| --- | --- | --- | --- | --- | --- | --- | --- | --- |
|  |  |  | YES | NO |  | Mean (SD) | Min | Max |
| Normal fetuses |  |  |  |  |  |  |  |  |
|  | 45-54 | 73 | 12(16.44%) | 61(83.56%) |  | 0.52(0.08) | 0.40 | 0.60 |
|  | 55-64 | 221 | 17((7.69%) | 204(92.31%) |  | 0.49(0.07) | 0.35 | 0.60 |
|  | 65-74 | 95 | 2(2.11%) | 93(97.89%) |  | 0.60(0) | 0.60 | 0.60 |
|  | 75-84 | 17 | 0 | 17(100%) |  | - | - | - |
|  | Total | 406 | 31(7.64%) | 375(92.36%) |  | 0.51(0.07) | 0.35 | 0.60 |
| Cleft lip and palate | |  |  |  |  |  |  |  |
|  | Total | 20 | 20(100%) | 0 |  | 1.55(0.68) | 0.80 | 3.10 |

CRL, crown-rump length; MG, maxillary gap.
